# Improving the Quality of Life in Patients With Hypohidrotic Ectodermal Dysplasia: A Holistic Approach

**DOI:** 10.7759/cureus.59847

**Published:** 2024-05-07

**Authors:** Harshitha Reddy, Anjalee Chiwhane, Manjeet Kothari, Yash Kashikar, Bhushan Madke

**Affiliations:** 1 Internal Medicine, Jawaharlal Nehru Medical College, Datta Meghe Institute of Higher Education and Research, Wardha, IND; 2 Dermatology, Jawaharlal Nehru Medical College, Datta Meghe Institute of Higher Education and Research, Wardha, IND; 3 Dermatology, Venereology, and Leprosy, Jawaharlal Nehru Medical College, Datta Meghe Institute of Higher Education and Research, Wardha, IND

**Keywords:** genetic disorder, hypotrichosis, hypodontia, anhidrosis, hypohydrotic ectodermal dysplasia, hypohidrosis

## Abstract

Hypohidrotic ectodermal dysplasia (HED), often referred to as Christ-Siemens-Touraine syndrome, is an uncommon inherited genetic disorder characterized by irregularities in structures derived from the ectoderm, such as skin, hair, nails, teeth, and sweat glands. Common manifestations include thin hair, absent teeth (hypodontia) often pointed in shape, and diminished ability to sweat (hypohidrosis). Changes in the ectodysplasin A (EDA) gene are associated with the development of HED. Addressing this condition requires an integrated, interdisciplinary strategy to ensure the best possible support for individuals impacted. This case highlights the significance of early detection, collaborative care, and targeted interventions in managing HED. Continued research is crucial for creating novel therapies and enhancing life quality for those living with this rare condition. Here, we discuss a 22-year-old male patient displaying features such as hypodontia, sparse hair (hypotrichosis), irregular beard growth, a nasal deformity, and an inability to sweat (anhidrosis), which is associated with increased body temperature.

## Introduction

Ectodermal dysplasia represents a group of uncommon inherited conditions, with an estimated prevalence of around 7 per 100,000 births. Atypical development of ectodermal tissues, such as hair, nails, teeth, sweat glands, sebaceous glands, the lining of the eyes, and elements of the nervous system, characterizes this category of illnesses, each of which is distinct both clinically and genetically [1]. This condition presents with a spectrum of clinical manifestations, primarily affecting the development of these structures, leading to distinctive physical features. While the condition is mainly inherited in an X-linked recessive manner, there have been reports of autosomal recessive and autosomal dominant forms.

Perabo and colleagues propose that the earliest recorded observation of ectodermal dysplasia could date back to 1792, attributed to the work of Danz [1]. In 1838, Wedderburn described ectodermal dysplasia in a letter to Charles Darwin, recounting a case involving 10 male family members of Hindu descent. The term "Inherited Ectodermal Dysplasia" was first introduced by Weech in 1929. He also suggested the descriptor "Anhidrotic" to characterize those lacking sweating ability. However, Felsher modified this term to "Hypohidrotic" in 1944, as it was believed that individuals with hypohidrotic forms still possess some sweat glands [2].

Critical characteristics of hypohidrotic ectodermal dysplasia (HED) encompass hypotrichosis (scanty hair), hypodontia (absence of teeth), and hypohidrosis (diminished sweating capacity). These symptoms can vary in their occurrence among those affected. Such conditions pose considerable difficulties in dental hygiene, body temperature management, and social engagement, significantly impacting the life experiences of both the patients and their relatives [3]. Despite its rarity, comprehending the clinical spectrum, genetic underpinnings, and management approaches for HED is imperative for early diagnosis, appropriate intervention, and supportive care.

## Case presentation

A 22-year-old male presented at the hospital exhibiting symptoms of absence of sweating, excessive heat intolerance, dryness of the eyes, and peg-shaped teeth with a reduced number of teeth since birth. The patient also reported a history of recurrent fevers since childhood, with no associated cranial nerve palsies or meningitis. Notably, the patient's parents had a second-degree consanguineous marriage, although neither they nor the patient's siblings displayed any similar abnormalities. Vital signs and systemic examination were within normal limits.

**Figure 1:**
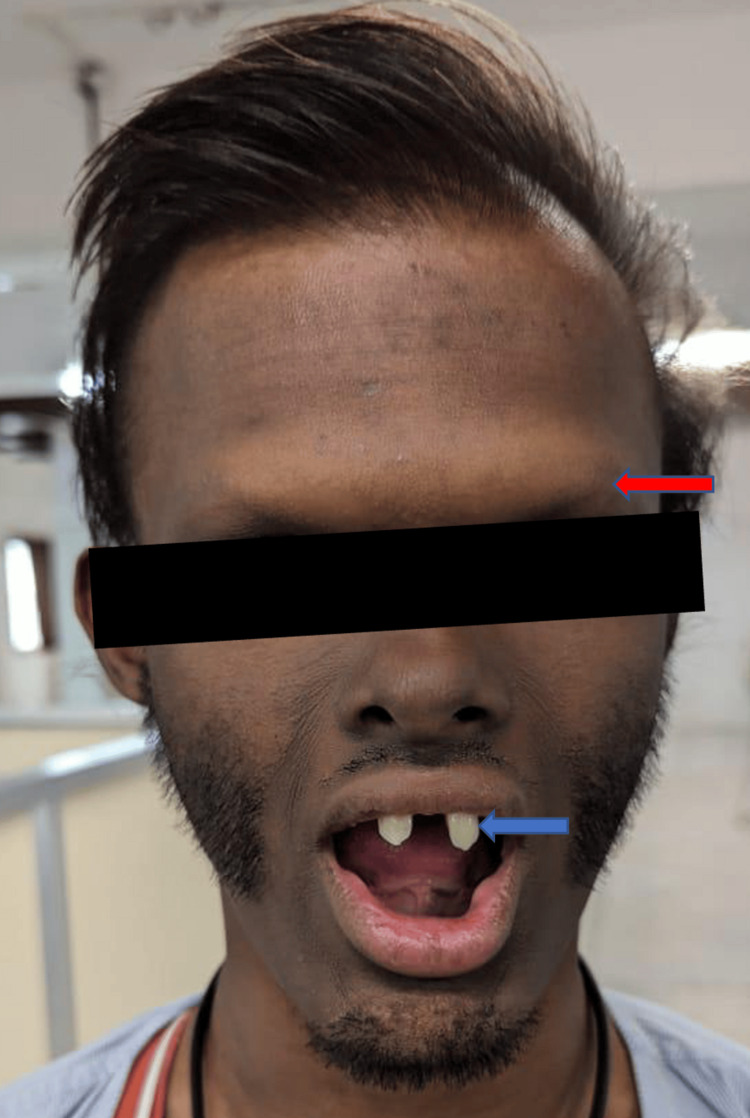
Prominent supra-orbital ridge with the absence of eyebrows (red arrow) and peg-shaped teeth (blue arrow)

Upon general examination, there is a prominent supra-orbital ridge and no eyebrows. In the oral cavity, peg-shaped teeth were observed, while the remaining teeth were absent (Figure 1).

**Figure 2:**
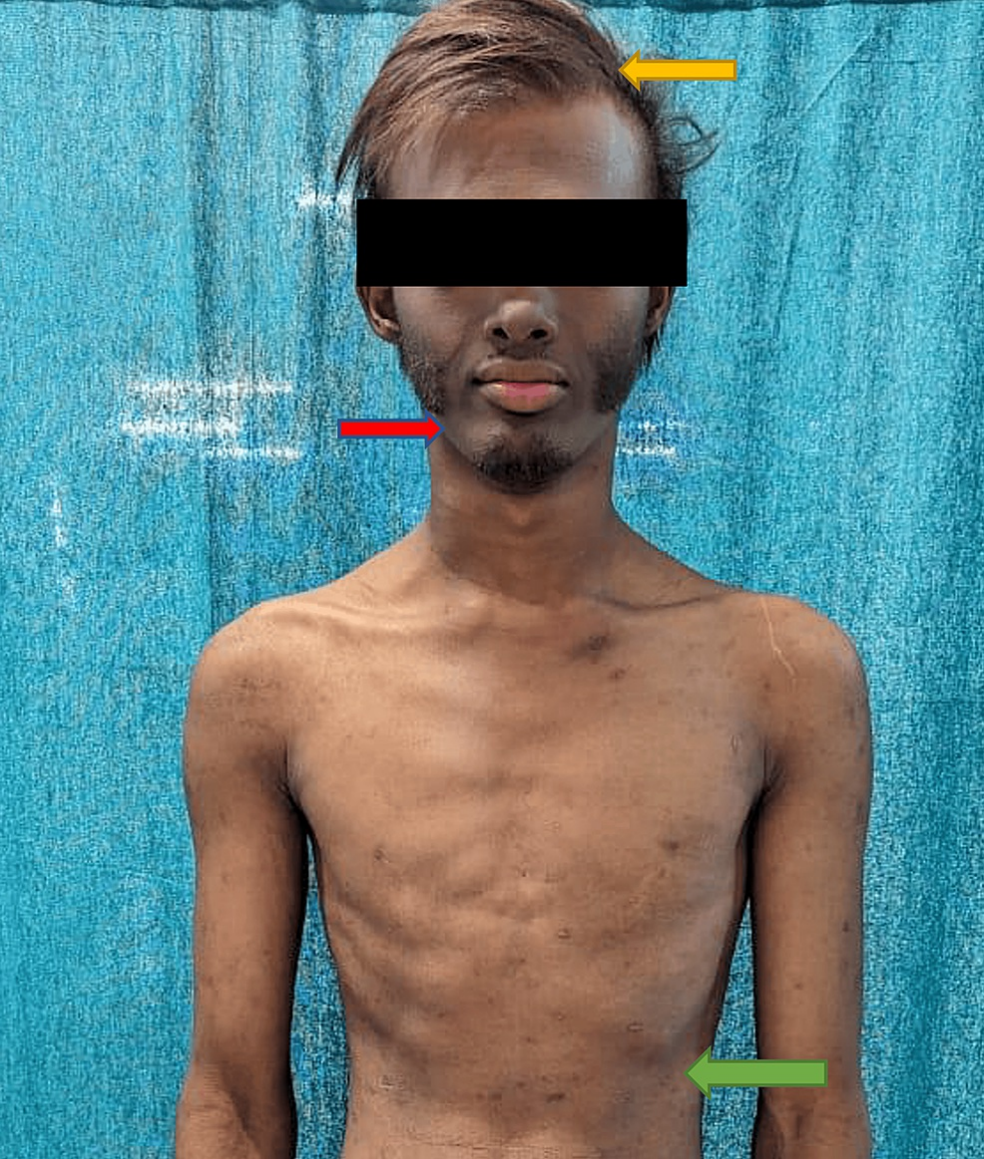
Lusturless hair (yellow arrow), peculiar beard distribution which is absent over the mandible (red arrow), and dry skin over the trunk (green arrow)

The beard distribution was peculiar, with absence over the mandible and dry skin over the trunk (Figure 2).

All laboratory tests are listed in Table 1.

**Table 1:** Laboratory investigations of the patient

Laboratory tests	Patient labs	Reference value
Hemoglobin	12.4	12-16 g/dl
White blood count	8220	4200-10100/cumm
Platelets	4,27,000	1,50,000-4,50,000/cumm
RBC count	4.32	4.2-5.5 million/cumm
Hematocrit	36.3	35-52%
Mean corpuscular volume	79.6	79-100 fL
Mean corpuscular hemoglobin	28.9	27-34 pg
Mean corpuscular hemoglobin concentration	32.9	31-36 gm/dl
Neutrophils	64	42-74%
lymphocytes	22	21-46%
Eosinophils	5.4	1-5%
Monocytes	8.4	2-8%
Basophils	0.1	1%
Urea	14.5	9-18 mg/dl
Creatinine	1	0.4-1.4 mg/dl
Sodium	138	136-144 mg/l
Potassium	4.4	3.6-5.0 mg/l
Alkaline phosphatase	67	34-120 units/liter
Alanine transaminase	12.7	<50 units/liter
Aspartate transaminase	13.1	16-56 units/liter
Serum protein	8.48	6.4-8.5 gm/dl
Serum albumin	3.8	3.6-5.2 gm/dl
Total bilirubin	0.20	0.2-1.2 mg/dl
Serum globulin	7.47	2.2-3.6 gm/dl
Thyroid-stimulating hormone	2.8	0.48-4.8 µIU/ml
Free T3	3.2	2.64-5.12 pg/ml
Free T4	1.3	0.54-2.40 ng/dl

**Figure 3:**
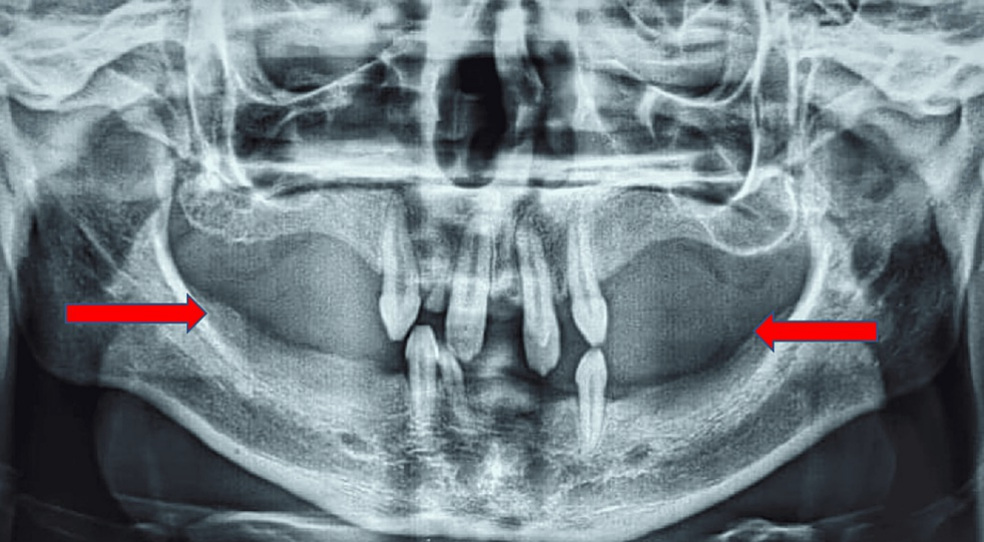
Orthopantomogram (OPG) was taken which revealed conical-shaped teeth and multiple missing teeth (red arrows)

An orthopantomogram (OPG) indicated the absence of several permanent and deciduous teeth. This imaging finding is consistent with the dental anomalies often associated with HED (Figure 3).

**Figure 4:**
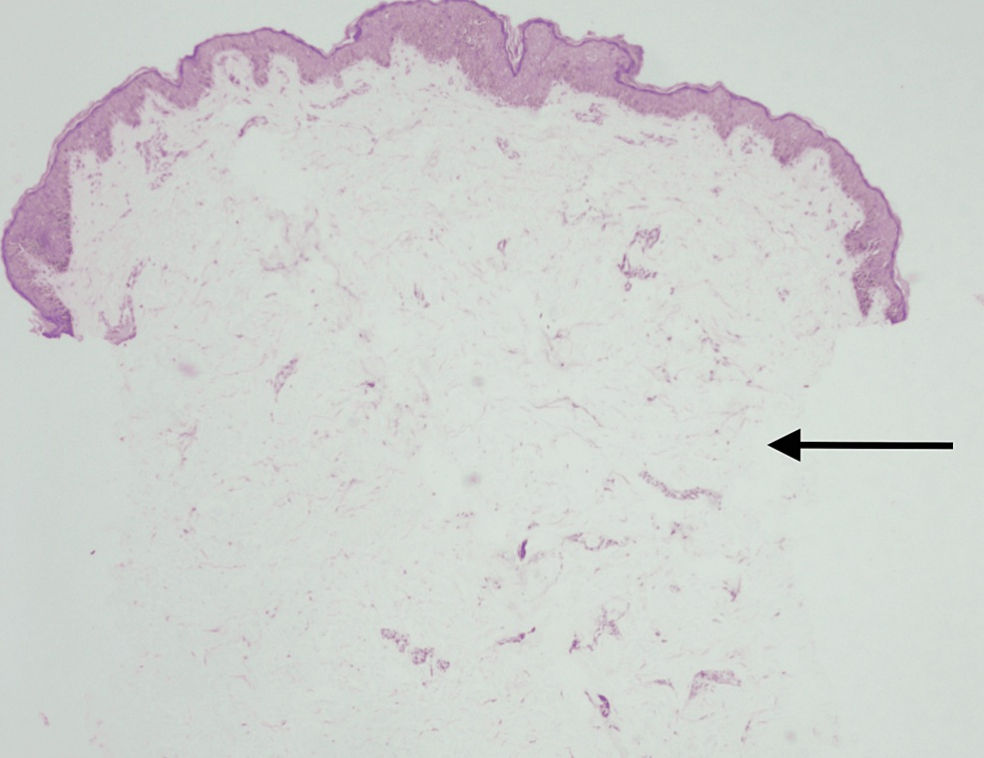
Histopathology examination (H&E, 40x magnification) of the skin revealed the absence of sweat glands (black arrow) H&E: hematoxylin and eosin stain

Histopathology examination (H&E, 40x magnification) of the skin revealed the absence of sweat glands (Figure 4).

The patient received a topical application of artificial tears to alleviate his dry eye symptoms. He was also counseled to maintain a cool environment in his living space, utilize a cooler or air conditioner, and wear loose cotton clothing to manage his heat intolerance. Although he was advised to consider undergoing artificial denture implantation to address his dental issues, he expressed reluctance. Given the genetic nature of his disorder and its lack of a cure, the patient was informed about the importance of genetic counseling before marriage and family planning. It was emphasized that seeking genetic counseling when considering marriage would be essential for informed decision-making regarding potential risks to offspring. Follow-up care will include continued genetic counseling as the patient decides about his future relationships and family planning.

## Discussion

Ectodermal dysplasia refers to various genetic conditions, each marked by fundamental developmental defects in at least two types of tissue originating from the embryonic ectoderm. In 1982, Freire-Maia and Pinheiro established an initial classification scheme for ectodermal dysplasia, organizing it according to the existence or nonexistence of irregularities in hair, teeth, nails, and sweat gland activity [3]. Subsequently, Nelson expanded this classification into five distinct groups: Hypohydrotic (anhydrotic), hydrotic (Clouston’s syndrome), ectrodactyly-ectodermal dysplasia-clefting (EEC) syndrome, Rapp-Hodgkin Syndrome, and Robinson’s disease [4]. The genetic variations responsible for these conditions, including ectodysplasin A (EDA), ectodysplasin A receptor (EDAR), EDAR associated via death domain (EDARADD), and WNT10A, follow autosomal dominant, autosomal recessive, and X-linked inheritance patterns. It is seen that males with hemizygous HED, who inherit the condition via X-linked transmission, often show more uniform and pronounced symptoms in contrast to females with heterozygous X-linked hypohidrotic ectodermal dysplasia (XLHED) [5].

Oral features such as missing teeth (hypodontia or anodontia) and teeth that are cone-shaped are frequently observed in those with ectodermal dysplasia. Prompt dental care is essential, encompassing treatments from basic repairs to complete dentures, particularly for children older than seven years. Dental implants and prostheses can greatly enhance mastication function and aesthetic appeal [6].

Caring for those with ectodermal dysplasia requires special attention to their inability to tolerate heat, especially during conditions such as fever or when engaging in physical activities, and their increased risk of respiratory infections. Recommended measures include ensuring the availability of chilled water, using air conditioning, dressing in breathable fabrics, and limiting exposure to direct sunlight [7]. Recent approaches to encourage hair regrowth in patients with ectodermal dysplasia include applying a 3% minoxidil solution. Emollient creams are the treatment of choice for dry skin and persistent dermatitis. Intensive wound management and topical and systemic antibiotics are critical in averting subsequent infections [7,8].

In particular, advancements in the molecular knowledge of XLHED have paved the way for specialized treatments such as EDI200. XLHED is caused by mutations in the ectodysplasin gene, which result in a shortage of the ectoderm signal protein EDA-A1. EDI200 acts as a synthetic analog of EDA-A1, stimulating the EDA-A1/EDAR signaling pathway, which is essential for developing ectodermal structures. Studies in preclinical settings have demonstrated encouraging changes in phenotype with EDI200 treatment, suggesting it may benefit those with XLHED [9,10]. Further research into the molecular processes underlying ectodermal dysplasia is anticipated to produce developments that might improve the quality of life for those afflicted with this disorder.

## Conclusions

HED is a rare genetic disorder characterized by distinct clinical features that affect various ectodermal structures. Timely recognition and diagnosis are paramount for initiating appropriate management and providing genetic counseling. Additionally, patient and parental education regarding the nature of the disease is crucial. Addressing the patient's primary symptoms, such as dry skin, dental abnormalities, and heat intolerance, is essential. Psychological counseling and genetic counseling should also be offered to the patient and their family members to address emotional and hereditary concerns. This case report aims to raise awareness among healthcare professionals about HED and highlight the significance of early diagnosis and multidisciplinary management.

## References

[1] Deshmukh S, Prashanth S (2012). Ectodermal dysplasia: a genetic review. Int J Clin Pediatr Dent.

[2] Suprabha BS (2002). Hereditary ectodermal dysplasia: a case report. J Indian Soc Pedod Prev Dent.

[3] Pinheiro M, Freire-Maia N (1994). Ectodermal dysplasias: a clinical classification and a causal review. Am J Med Genet.

[4] Cambiaghi S, Restano L, Pääkkönen K, Caputo R, Kere J (2000). Clinical findings in mosaic carriers of hypohidrotic ectodermal dysplasia. Arch Dermatol.

[5] Wright JT, Grange DK, Fete M (1993). Hypohidrotic ectodermal dysplasia. GeneReviews® [Internet].

[6] Agarwal N, Kumar D, Anand A, Bahetwar SK (2016). Dental implants in children: a multidisciplinary perspective for long-term success. Natl J Maxillofac Surg.

[7] Brengelmann GL, Freund PR, Rowell LB, Olerud JE, Kraning KK (1981). Absence of active cutaneous vasodilation associated with congenital absence of sweat glands in humans. Am J Physiol.

[8] Kumar S, Verma A (2013). Holocord syrinx presenting as hemi anhidrosis. Indian Dermatol Online J.

[9] Reyes-Reali J, Mendoza-Ramos MI, Garrido-Guerrero E, Méndez-Catalá CF, Méndez-Cruz AR, Pozo-Molina G (2018). Hypohidrotic ectodermal dysplasia: clinical and molecular review. Int J Dermatol.

[10] Schneider H, Schweikl C, Faschingbauer F, Hadj-Rabia S, Schneider P (2023). A causal treatment for X-linked hypohidrotic ectodermal dysplasia: long-term results of short-term perinatal ectodysplasin A1 replacement. Int J Mol Sci.

